# Epigenome-wide DNA Methylation Association Study of CHIP Provides Insight into Perturbed Gene Regulation

**DOI:** 10.21203/rs.3.rs-4656898/v1

**Published:** 2024-07-16

**Authors:** Daniel Levy, Sara Kirmani, Tianxiao Huan, Joseph Van Amburg, Roby Joehanes, Md Mesbah Uddin, Ngoc Quynh Nguyen, Bing Yu, Jennifer Brody, Myriam Fornage, Jan Bressler, Nona Sotoodehnia, David Ong, Fabio Puddu, James Floyd, Christie Ballantyne, Bruce Psaty, Laura Raffield, Pradeep Natarajan, Karen Conneely, April Carson, Leslie Lange, Kendra Ferrier, Nancy Heard-Costa, Joanne Murabito, Alexander Bick

**Affiliations:** Framingham Heart Study, Framingham, MA, 01702, USA; Population Sciences Branch, Division of Intramural Research, National Heart, Lung, and Blood Institute, National Institutes of Health; Framingham Heart Study, Framingham, MA, 01702, USA; Population Sciences Branch, Division of Intramural Research, National Heart, Lung, and Blood Institute, National Institutes of Health, Bethesda; The Framingham Heart Study; Division of Genetic Medicine, Department of Medicine, Vanderbilt University Medical Center; National Heart Lung & Blood Institute; Broad Institute of MIT and Harvard; University of Texas Health Science Center at Houston; University of Texas Health Science Center at Houston; Cardiovascular Health Research Unit; 1. Institute of Molecular Medicine, McGovern Medical School, The University of Texas Health Science Center 2. Human Genetics Center, Department of Epidemiology, School of Public Health; School of Public Health, University of Texas Health Science Center at Houston; University of Washington; Division of Genetic Medicine, Department of Medicine, Vanderbilt University Medical Center, Nashville, TN, 37232, USA; biomodal; University of Washington; Baylor College of Medicine; Cardiovascular Health Research Unit; University of North Carolina at Chapel Hill; Broad Institute of Harvard and Massachusetts Institute of Technology; Emory University School of Medicine; University of Mississippi Medical Center; Division of Biomedical Informatics and Personalized Medicine; University of Colorado; Boston University Chobanian & Avedisian School of Medicine; Section of General Internal Medicine, Boston University Chobanian & Avedisian School of Medicine; Vanderbilt University Medical Center

## Abstract

With age, hematopoietic stem cells can acquire somatic mutations in leukemogenic genes that confer a proliferative advantage in a phenomenon termed “clonal hematopoiesis of indeterminate potential” (CHIP). How these mutations confer a proliferative advantage and result in increased risk for numerous age-related diseases remains poorly understood. We conducted a multiracial meta-analysis of epigenome-wide association studies (EWAS) of CHIP and its subtypes in four cohorts (N=8196) to elucidate the molecular mechanisms underlying CHIP and illuminate how these changes influence cardiovascular disease risk. The EWAS findings were functionally validated using human hematopoietic stem cell (HSC) models of CHIP. A total of 9615 CpGs were associated with any CHIP, 5990 with DNMT3A CHIP, 5633 with TET2 CHIP, and 6078 with ASXL1 CHIP (P <1×10^−7^). CpGs associated with CHIP subtypes overlapped moderately, and the genome-wide DNA methylation directions of effect were opposite for TET2 and DNMT3A CHIP, consistent with their opposing effects on global DNA methylation. There was high directional concordance between the CpGs shared from the meta-EWAS and human edited CHIP HSCs. Expression quantitative trait methylation analysis further identified transcriptomic changes associated with CHIP-associated CpGs. Causal inference analyses revealed 261 CHIP-associated CpGs associated with cardiovascular traits and all-cause mortality (FDR adjusted p-value <0.05). Taken together, our study sheds light on the epigenetic changes impacted by CHIP and their associations with age-related disease outcomes. The novel genes and pathways linked to the epigenetic features of CHIP may serve as therapeutic targets for preventing or treating CHIP-mediated diseases.

## Introduction

A hallmark of aging is the accumulation of somatic mutations in dividing cells. The vast majority of these mutations do not affect cell fitness. In rare circumstances, however, a mutation can arise that confers a selective fitness advantage, culminating in its expansion relative to other cells. In the hematopoietic system, this process is termed clonal hematopoiesis (CH). Individuals with CH are at increased risk for the development of hematologic malignancies.^1^ A subset of CH is driven by pathogenic mutations in myeloid malignancy-associated genes, which is termed CH of indeterminate potential (CHIP) and has been shown to be associated with hematologic cancers, cardiovascular disease (CVD), chronic obstructive pulmonary disease, and mortality, among other conditions.^3–5^

The prevalence of CHIP increases with advancing age.^3, 6–8^ In a whole genome sequencing (WGS) study from the NHLBI Trans-Omics for Precision Medicine (TOPMed) program that included ~ 100,000 individuals across 51 separate studies, large CHIP clones were found to be uncommon (< 1%) in individuals younger than 40 years of age and increased to 12% in those aged 70–89 and 20% in those aged 90 years and older.^6^ This age-dependent pattern was consistent across CHIP driver genes^6^ and has been observed in other studies.^3, 7, 8^

DNA methylation (DNAm), the addition of a methyl group to a cytosine followed by a guanosine (CpG) in DNA, is an epigenetic modification that reflects age and environmental exposures. The gene products of the three most frequently mutated CHIP driver genes, *DNMT3A*, *TET2*, and *ASXL1*, are epigenetic regulators^6^. DNMT3A (DNA-methyltransferase 3A) is a methyltransferase that catalyzes the transfer of methyl groups to CpG sites and catalyzes *de novo* DNA methylation.^9^ Conversely, TET2 (ten-eleven translocation-2) is a DNA demethylase that catalyzes the conversion of 5-methylcytosine to 5-hydroxymethylcytosine, one of the steps leading to eventual demethylation of CpG sites.^10^ ASXL1 (ASXL transcriptional regulator 1) is involved in histone modification.^11^ Its function in CHIP remains relatively unknown.^12^

CHIP has been shown to be associated with global DNAm changes, particularly for the *DNMT3A* and *TET2* CHIP driver gene mutations.^13^ A previous epigenome-wide association study (EWAS) of CHIP in 582 Cardiovascular Health Study (CHS) participants, with replication in 2655 Atherosclerosis Risk in Communities (ARIC) participants, revealed several thousand CpG sites associated with CHIP and its two major CHIP driver genes, *DNMT3A* and *TET2*.^13^ *DNMT3A* and *TET2* CHIP were also found to have directionally opposing DNAm signatures: *DNMT3A* CHIP mutations were associated with hypomethylation of CpGs, whereas *TET2* CHIP was associated with hypermethylation of CpGs, consistent with the canonical regulatory functions of DNMT3A and TET2 elucidated in murine and human model systems.^14–16^

Despite the wealth of information from the previous EWAS of CHIP^13^, several limitations and knowledge gaps remain. These include the need to use larger sample sizes to enable analyses of less prevalent CHIP driver gene mutations such as *ASXL1*, explore downstream functions and pathways influenced by mRNA expression for any CHIP and CHIP subtypes, and identify underlying molecular mechanisms linking CHIP to CVD.

**Figure 1:**
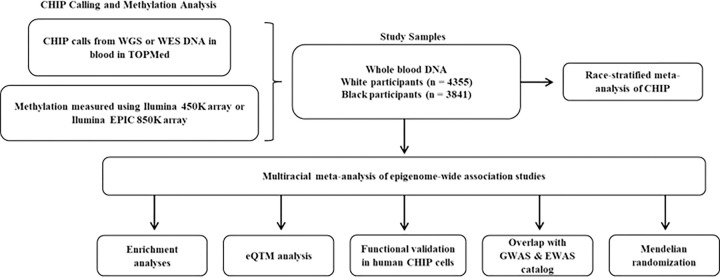
Overview of Study Design

To address these knowledge gaps, we conducted a multiracial meta-analysis of separate EWAS of CHIP in four independent cohort studies (N = 8196; 462 with any CHIP, 261 *DNMT3A*, 84 *TET2*, and 21 with *ASXL1* CHIP) along with analysis of the associations of CHIP-related CpGs with downstream gene expression. We expanded upon the previous EWAS of CHIP study^13^ by adding two cohorts – the Framingham Heart Study (FHS) and the African-American Jackson Heart Study (JHS) – in addition to the ARIC and CHS cohorts. The EWAS findings were functionally validated using human hematopoietic stem cell (HSC) models of CHIP. Expression quantitative trait methylation (eQTM) analysis identified gene expression changes associated with CHIP-associated CpGs. Causal inference analysis using two-sample Mendelian randomization (MR) was performed to gain insight into the molecular mechanisms linking CHIP to CVD. A flowchart of the study design is shown in Fig. 1.

## Results

### Clinical Characteristics of Study Participants

The baseline characteristics of FHS, JHS, CHS, and ARIC participants included in this investigation are presented in Table 1. The mean age at the time of blood draw for whole-genome sequencing (WGS) was 57, 56, and 58 for FHS, JHS, and ARIC, respectively. Participants from CHS were considerably older, with a mean age of 74 years. All four cohorts had more women than men (54–63% women). Overall, CHIP mutations with a variant allele frequency (VAF) ≥ 2% were present in 5% (166/3295) of participants in FHS, 4% (68/1664) in JHS, 5% (142/2655) in ARIC, and 15% (86/582) in CHS. Consistent with previous reports^6^, the three most frequently mutated CHIP driver genes across all cohorts were *DNMT3A, TET2*, and *ASXL1*. Eighty percent of individuals with CHIP demonstrated expanded CHIP clones with VAF > 10%.

### Epigenome-wide Association Analysis

#### Race-specific analysis

Race was classified based on self-report. In the race-stratified analysis, we identified 2843 CpGs associated with any CHIP, 758 with *DNMT3A*, 4735 with *TET2* CHIP in White participants and 5498 with any CHIP, 5065 with *DNMT3A*, and 290 with *TET2* CHIP in Black participants at Bonferroni-corrected *P* < 1 × 10^−7^ (Supplementary Tables 1–6). 1290, 675, and 254 CHIP-associated CpG sites were shared between White and Black participants at the Bonferroni-corrected threshold, with concordant directions of effect for any CHIP, *DNMT3A*, and *TET2* CHIP, respectively.

#### Multiracial analysis

In a multiracial, meta-EWAS of CHIP, 9615 CpGs were associated with any CHIP, and 5990, 5633, and 6078 CpGs were associated with *DNMT3A* CHIP, *TET2* CHIP, and *ASXL1* CHIP, respectively (at Bonferroni-corrected *P* < 1 × 10^−7^). The top ten CpGs for any CHIP and for each of the three CHIP driver genes are shown in Table 2. A full list of CpG signatures and their directions of effect are reported in Supplementary Tables 7–10. There was minimal to moderate overlap of CpGs associated with *DNMT3A*, *TET2*, and *ASXL1*; 429, 904, and 1088 CpG sites were shared between *DNMT3A* and *TET2*, *DNMT3A* and *ASXL1*, and *TET2* and *ASXL1*, respectively.

**Figure 2:**
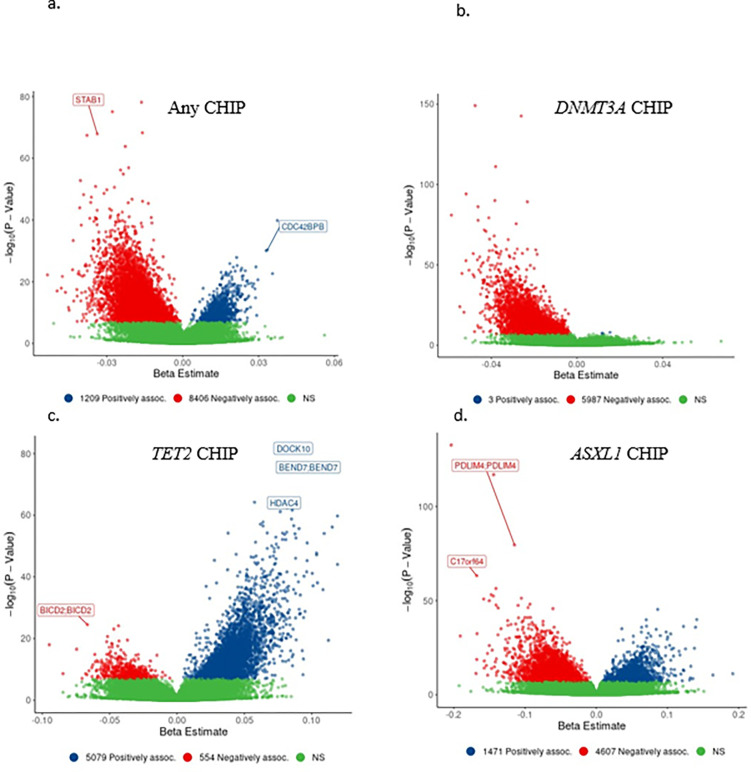
Genome-wide Directions of Effect of any CHIP and CHIP subtypes a-d. Volcano plots with the effect size ( ) and −log_10_(P) from the multiracial meta EWAS of (a.) any CHIP, (b.) *DNMT3A* CHIP, (c.) *TET2* CHIP, and the EWAS in FHS of (d.) *ASXL1* CHIP. Genes annotated to the CpG sites are shown.

We identified 5987 CpGs (~ 100%) associated with *DNMT3A* CHIP and 4607 CpGs (~ 76%) associated with *ASXL1* CHIP that showed decreased methylation (β < 0) (Fig. 2b and 2d). In contrast, 5079 CpGs (~ 90%) associated with *TET2* CHIP showed increased methylation (β > 0) (Fig. 2c). The vast majority of CpGs associated with CHIP were remote (> 1 Mb) from the driver gene including 5969/5990 (99.6%) for *DNMT3A*, 5632/5633 (~ 100%) for *TET2*, and 6070/6078 (99.9%) for *ASXL1*.

A sensitivity analysis was performed by excluding CHIP cases with VAF < 10%. The results are similar to the multiracial meta-EWAS of any CHIP and are provided in Supplementary Fig. 4 and Supplementary Tables 11–13. Approximately 78% of CpGs (7460/9615) in the meta-EWAS of any CHIP were re-identified in the sensitivity analysis, while 312 CpGs were newly identified.

### Engineered Human Hematopoietic Stem Cell Models of CHIP Validate EWAS Results

We sought to experimentally validate our multiracial meta-EWAS methylation findings with an in vitro model of CHIP. CHIP was modeled by introducing loss-of-function mutations in *DNMT3A, TET2*, and *ASXL1* in mobilized peripheral blood CD34 + hematopoietic cells, using CRISPR-Cas9.^17^ After seven days in culture, these cells were flow sorted to isolate a purified population of CD34^+^CD38^−^Lin^−^ cells. Following fluorescence-activated cell sorting, genomic DNA (gDNA) was extracted, and methylation was assayed using Biomodal EvoC (see Methods).^18^

**Figure 3:**
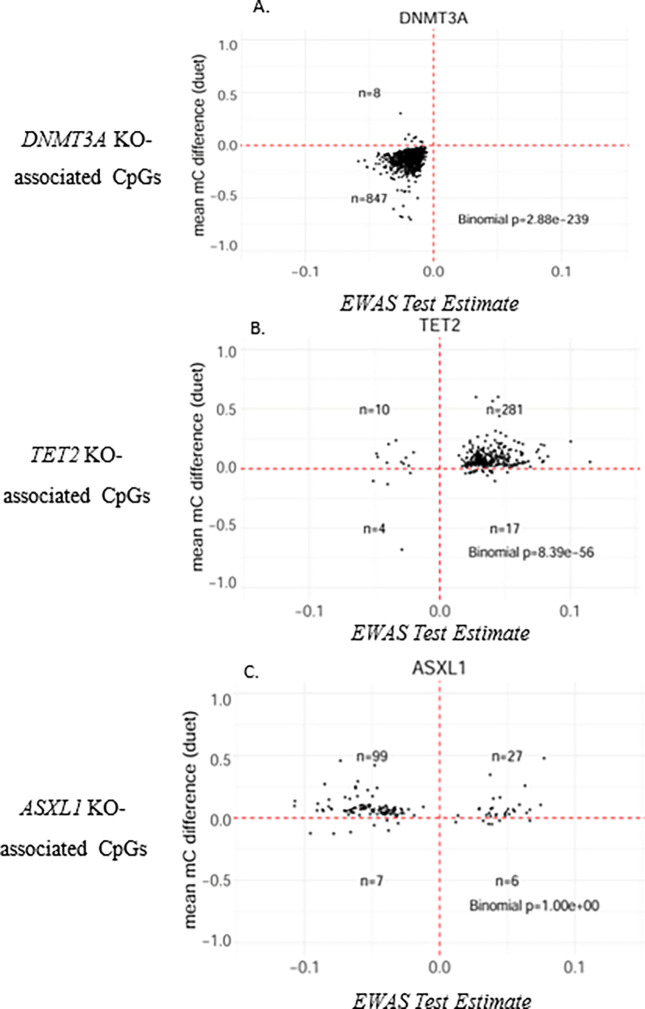
Functional Validation in CRISPR/Cas9-edited HSCs Modeling CHIP Dot plots of methylation status from −1.0 (no methylation) to 1.0 (complete methylation) seen in engineered primary cell cultures compared to correlation of EWAS results ranging from −0.1 to 0.1. Significance was determined with a binomial test. A) *DNMT3A*-associated CpG sites (n = 855 CpG sites) compared to *DNMT3A* engineered human stem cells(n=4). B) *TET2*-associated CpG sites (n = 312) compared to *TET2*-engineered human stem cells (n=4). C) *ASXL1*-associated CpG Sites (n = 139) compared to *ASXL1*-engineered human stem cells (n = 3).

The analysis focused on the subset of CpG sites that were significantly associated with CHIP in the EWAS data and nominally differentially methylated (*P* < 0.05) in the in vitro model of CHIP. When comparing CpG site subsets with their respective engineered cells, *DNMT3A*-associated CpG sites showed significant enrichment in *DNMT3A*-engineered cells (*P* < 2.88 × 10^− 239^) (Fig. 3A), while *TET2*-associated CpG sites were significantly enriched in TET2-engineered cells (*P* < 8.39 × 10^− 56^) (Fig. 3B), and *ASXL1*-engineered cells (*P* < 1.65 × 10^− 14^) (Supplementary Fig. 5). *ASXL1*-associated CpG sites showed no significant hits in *ASXL1*-engineered cells (Fig. 3C), but a slight trend in *TET2*-engineered cells (Supplementary Fig. 5). The any CHIP-associated CpG sites were significantly enriched in *DNMT3A*-engineered primary cells only (*P* < 9.29 × 10^− 121^), unlike *TET2* and *ASXL1* (Supplementary Fig. 5).

### Association of DNA Methylation with Gene Expression and Pathway Analyses

To investigate the functional consequences of CHIP-associated CpGs, we performed gene ontology (GO) and pathway enrichment analysis for genes harboring CHIP-associated CpGs. For any CHIP, *DNMT3A* CHIP, and *ASXL1* CHIP the enriched GO terms related to broad cellular developmental and organismal processes, while for *TET2* CHIP the top GO terms related to regulatory and inflammatory processes (Supplementary Tables 14–17).

To understand how differentially methylated CpGs in association with CHIP might alter cellular function, we identified gene expression changes associated with CHIP-linked CpGs. We analyzed the associations of CHIP-associated CpGs with changes in *cis* gene expression (expressed gene [eGene] within 1 Mb of CpG) in 2115 FHS participants whose DNA methylation data and whole-blood RNA-seq data were available. At *P* < 1 × 10^−7^, we identified 1658 unique, significant *cis* CpG-transcript pairs for any CHIP, 1059 for *DNMT3A* CHIP, 1202 for *TET2* CHIP, and 1003 for *ASXL1* CHIP (Supplementary Tables 18–21 provide the full expression quantitative trait methylation (eQTM) results).^19^ Across all CHIP cases as well as for CHIP subtypes, the majority of the expressed genes (eGenes) associated with a CHIP-associated CpG were enriched in pathways related to various immune functions and cellular processes at *P* < 0.05 (Supplementary Tables 22–25). The GO enrichment results, however, were not significant after correction for multiple testing.

### Association of DNA Methylation with Genetic Variants and Mendelian Randomization Analysis

*Cis*-methylation quantitative trait loci (*cis*-mQTL) – genetic loci that are significantly associated with CpG methylation levels and located within 1 Mb of their associated CpG – linked 8642 CpGs associated with any CHIP and CHIP subtypes to GWAS Catalog traits/diseases.^20, 21^ Of the *cis*-mQTL variants, a subset were associated with clonal hematopoiesis traits, particularly myeloid clonal hematopoiesis and the number of clonal hematopoiesis mutations (Supplementary Table 26).

Additionally, enrichment tests of CHIP-associated CpG sites with EWAS catalog traits^22^ were performed across 4023 traits using a significance threshold of 1.24 × 10^−5^ (0.05/4023) (Supplementary Table 27). For any CHIP, *DNMT3A* CHIP, *TET2* CHIP, and *ASXL1* CHIP, the top outcomes reflected CpG sites related to age/aging, alcohol consumption, smoking, and multiple CVD-related traits including body mass index (BMI), type II diabetes, and fasting insulin. In support of previous studies reporting *ASXL1* CHIP enrichment among smokers^23, 24^, 24% (1462/6078) of *ASXL1* CHIP-associated CpGs overlapped with smoking-associated CpGs.

Two-sample MR analysis of CHIP-associated CpGs (as exposures) with *cis*-mQTLs as the instrumental variables in relation to CVD-related traits and mortality (as outcomes) was performed to infer whether differential methylation at CHIP-associated CpGs may causally influence the outcomes. The significantly associated CpGs for any CHIP and for the three CHIP driver genes were tested for causal associations with 22 traits, including all-cause mortality, BMI, LDL cholesterol, hypertension, diabetes, CVD, and smoking. The top 20 CpGs and annotated genes for each trait are reported in Table 3 (Supplementary Table 28 displays the full MR results). 261 CHIP-associated, differentially methylated CpG sites were identified that were putatively causally associated with CVD-related traits and/or all-cause mortality, including eight CpGs for myocardial infarction (MI) (e.g., cg11879188 (*ABO*), β_MR_ =−0.99, P_MR_ = 4.8 × 10^−18^), 108 CpGs for blood pressure (e.g., cg20305489 (*SEPT9*), β_MR_ = 10, P_MR_ = 1.7 × 10^−31^), 86 CpGs for lipid traits (e.g., cg11250194 (*FADS2*), β_MR_ =−0.89, P_MR_ = 2.0 × 10^−33^), and two CpGs for mortality (e.g., cg08756033 (*C13orf33*), β_MR_ = 0.016, P_MR_ = 1.3 × 10^−4^). 53 CpGs were associated with more than one trait. For example, cg11879188 is annotated to the ABO gene and is associated with seven traits, including diastolic blood pressure (β_MR_ = 2.7, P_MR_ = 1.9 × 10^−23^), MI (β_MR_ =−0.99, P_MR_ = 4.8 × 10^−18^), and triglycerides (β_MR_ = 0.20, P_MR_ = 2.0 × 10^−9^).

## Discussion

We report the results of a multiracial meta-EWAS of CHIP and identified thousands of CpG sites across the genome that are significantly associated with any CHIP and with *DNMT3A*, *TET2*, and *ASXL1* CHIP. Of note, the vast majority of the CpGs were trans-relative to the CHIP driver gene. This appears to be consistent with the functions of *DNMT3A*, *TET2*, and *ASXL1* in globally altering DNA methylation levels of CpG sites genome wide, as seen in the EWAS of each of the three CHIP driver genes, where the significantly associated CpGs were numerous and located diffusely across the genome. The methylomic signatures of CHIP and CHIP driver genes were experimentally validated with human-engineered CHIP cells. Downstream analyses were conducted to assess whether these alterations in DNA methylation levels may be causally associated with CVD-related outcomes and all-cause mortality. Causal inference analyses using two-sample MR revealed evidence of a possible causal role of CHIP-associated CpGs in various CVD-related traits and all-cause mortality.

For the experimental validation of our meta-EWAS results, any CHIP-associated CpG sites were significantly enriched in *DNMT3A*-engineered cells, which was expected given the overwhelming predominance of *DNMT3A* CHIP among total CHIP cases reported in our study and several others^3, 6, 13^. Interestingly, *TET2*-associated CpG sites were enriched in *ASXL1*-engineered cells. This finding is consistent with the substantial CpG overlap (~1000 shared CpGs) between *TET2* and *ASXL1* CHIP from the meta-EWAS and suggests that the epigenetic regulators *TET2* and *ASXL1* impact several of the same genome regions and may lead to similar downstream consequences. Interestingly, *ASXL1*-associated CpGs showed no significant enrichment in the *ASXL1*-engineered cells, which may be due to the limited number of *ASXL1* CHIP cases in the EWAS.

Two-sample MR analysis identified 261 differentially methylated CpG sites that were putatively causally related to one or more CVD traits and/or all-cause mortality. For example, cg11250194 was putatively causally associated with four CVD-related cardiometabolic traits: LDL cholesterol, HDL cholesterol, triglycerides, and fasting glucose. Cg11250194 resides in the *FADS2* gene. It is hypomethylated, associated with *DNMT3A* CHIP ( =−0.022, P=1.6E-13), and replicated in the *DNMT3A* CHIP-engineered cells. The *FADS2* gene encodes the enzyme fatty acid desaturase 2 – the first rate-limiting enzyme for the biosynthesis of polyunsaturated fatty acids.^25^ A recent study found that cg11250194 (*FADS2*) was associated with Alternative Healthy Eating Index and that hypermethylation of this CpG was associated with lower triglyceride levels^26^. Based on our findings, hypomethylation of this diet-associated CpG may be linked to higher triglyceride levels, putatively increasing the risk for CVD. *FADS2* overexpression has also been found to promote clonal formation.^25^ Thus, *FADS2* may be an important gene connecting CHIP with diet. Of note, of the 30 CpGs associated with either Mediterranean-style Diet Score or Alternative Healthy Eating Index or both in a 2020 study by Ma et al. ^26^, 17 were CHIP-associated CpGs (~57%) identified from our multiracial meta-EWAS of CHIP. The substantial overlap between diet- and CHIP-associated CpGs is consistent with the hypothesis that an unhealthy diet may be associated with CHIP through epigenetic mechanisms.

Compared to a previously published EWAS of CHIP^13^ (N=3273, 228 CHIP cases), the present study has a substantially larger sample size (N=8196, 462 CHIP cases), including all the samples from the previous study. With the larger sample size of the present study, we identified 6687, 3524, and 4678 novel CpGs significantly associated with any CHIP and with the top two CHIP driver genes *DNMT3A* and *TET2*. Of the CpG sites identified from the previous EWAS study at *P* <1, a large proportion overlapped and have concordant directions of effect with CpGs from the multiracial meta-EWAS of CHIP at *P* <1:91% (2928/3217) for any CHIP, 89% (2466/2769) for *DNMT3A* CHIP, and 90% (955/1059) for *TET2* CHIP. This is expected, as almost half of the CHIP cases in our meta-EWAS of CHIP are from the previous EWAS of CHIP^13^. Additionally, to our knowledge, we provide the first EWAS of *ASXL1* CHIP and report thousands of novel *ASXL1* CHIP-associated CpGs. Through eQTM analysis that identified CpG-transcript pairs, the top eGenes in *ASXL1* CHIP relate to various immune processes, suggesting that dysregulated immune function, particularly among T cells, may contribute to *ASXL1* CHIP-related disease outcomes. This putative role of *ASXL1* CHIP in perturbing immune function, specifically T cell function, has been recently reported using an *ASXL1* CHIP conditional knock-in mouse model.^27^ Notably, several of the *ASXL1* CHIP-associated CpGs displayed putatively causal relations to CVD-related traits in MR analysis, including cg11879188 (in *ABO*).

While there are several strengths of our study, some limitations should be noted. A larger sample size is needed to examine less frequently mutated CHIP driver genes, such as *TP53, JAK2*, and *PPM1D*. Additionally, the reported putatively causal associations of CpGs with CVD outcomes and mortality were based on two-sample MR analysis; our findings warrant validation. Taken together, our study sheds light on the epigenetic changes linked to CHIP and CHIP subtypes and their associations with CVD-related outcomes. The novel genes and pathways linked to the epigenetic features of CHIP may serve as therapeutic targets for CHIP-related diseases. More broadly, our results provide insight into the molecular mechanisms underlying age-related diseases.

## Methods

### Study Cohorts

The Framingham Heart Study (FHS) is a prospective, observational community-based cohort investigating risk factors for CVD. For our discovery sample, DNAm was measured from FHS participants (N=3295) in the Offspring cohort (N=1860; Exam 8; years 2005–2008)^28^ and in the Third Generation cohort (N=1435; Exam 2; years 2008–2011).^29^ CHIP calls were based on whole-genome sequencing of whole blood DNA samples, the majority of which were from FHS Offspring participants at Exam 8 and Gen 3 participants at Exam 2 and temporally concordant with the time of DNAm profiling. All FHS participants self-identified as White at the time of recruitment.

The Jackson Heart Study (JHS) is an observational community-based cohort studying the environmental and genetic factors associated with CVD in African Americans. For our discovery sample, data were collected from 1664 JHS participants.^13^ DNAm was measured from the majority of JHS participants at visit 1, with a small subset at visit 2. CHIP calls were concurrent with DNAm profiling and based on whole-genome sequencing of whole blood DNA samples, where the majority were from visit 1 (years 2000–2004) and a subset from visit 2 (years 2005–2008).^13^ All JHS participants self-identified as Black or African American at the time of recruitment. No ancestry outliers were excluded, as inferred based on genetic similarity to reference panels. Similarity to the 1000G AFR reference panel varied by individual (study q1, median, q3 77.9% 84.3% 89.0%) in the methylation and WGS overlap dataset, using estimates from RFMix.

The Cardiovascular Health Study (CHS) is a population-based cohort study of risk factors for CVD in adults aged 65 or older.^2^ DNAm was measured from blood samples from participants in years 5 and 9, year 5, or year 9 only. CHIP calls were based on whole-genome sequencing of blood samples, where the majority were taken 3 years before or concurrently with the first DNAm measurement.^13^ CHS participants self-reported their race at the time of recruitment.

The Atherosclerosis Risk in Communities (ARIC) is a prospective, multiracial cohort study of risk factor and clinical outcomes of atherosclerosis.^30^ DNAm was measured from 2655 ARIC participants at visit 2 (1990–1992) or visit 3 (1993–1995). CHIP calls were based on whole exome sequencing of blood samples from visit 2 and visit 3.^13, 31^ ARIC participants self-identified their race at the time of recruitment.

### DNA Methylation Profiling

All the DNA samples were from whole blood. The four cohorts including FHS, JHS, CHS and ARIC, conducted independent laboratory DNAm measurements, quality control (including sample-wise and probe-wide filtering and probe intensity background correction; see additional file 1). DNA methylation was measured in FHS, CHS, and ARIC participants using Illumina Infinium Human Methylation-450 Beadchip (450K array) and in JHS participants using the Ilumina EPIC array as previously described^32, 33^.

### CHIP Calling

For the purposes of this investigation, CHIP was defined as a candidate driver gene mutation in genes that have been reported to be associated with hematologic malignancy, is present at a variant allele frequency (VAF) of at least 2% in peripheral blood, and is present in the absence of hematologic malignancy.^34^ CHIP was detected in FHS, JHS, and CHS from WGS blood DNA in the NHLBI Trans-Omics for Precision Medicine (TOPMed) consortium using the Mutect2 software as previously described.^6^ In ARIC, CHIP calls were based on whole exome sequencing of blood DNA using the same procedure.^6^ CHIP is defined as when an individual harbors at least one pre-specified deleterious insertion/deletion or single nucleotide variant in any of the 74 genes linked to myeloid malignancy at a variant allele frequency (VAF) ≥2%.^6^ TOPMed WGS samples were sequenced to a median depth of 40x, with the sequencing depth ranging from 30x-50x for a specific region. At this sequencing depth, CHIP can be reliably ascertained with a VAF >10% but CHIP variants with a VAF ≤10% are unable to be robustly captured.^6^ For a sensitivity analysis, ancestry-stratified and pooled ancestry meta-EWAS of any CHIP was performed using a more restrictive CHIP clone size of VAF >10% (See Supplementary Figure 4 and Supplementary Tables 11–13).

### Cohort-Specific EWAS

The correction of methylation data for technical covariates was cohort specific. Each cohort performed an independent investigation to select an optimized set of technical covariates (e.g., batch, plate, chip, row, and column), using measured or imputed blood cell type fractions, surrogate variables, and/or principal components. Most cohorts had previous publications using the same dataset for EWAS of different traits, such as EWAS of alcohol drinking and smoking. In this study, those cohorts used the same strategies as they did previously for correcting for technical variables, including batch effects. Linear mixed models were used to test the associations between CHIP status as the predictor variable and DNAm β values as the outcome variable. Information about cohort-specific models is available in Supplementary File 2.

### Meta-analysis

All analyses were contingent on self-reported Black or White race. Previous ancestry inference in these cohort studies^35^ suggests high genetic similarity of nearly all self-identified White participants to EUR reference panels (including 1000 Genomes). Self-identified Black participants have high but variable (average ~80% but may vary based on study and by study participant) genetic similarity to AFR reference panels and have some similarity to EUR reference panels as well. In some cases, extreme ancestry outliers may have been removed during study-specific QC. However, this has not been thoroughly documented in the data we received from participating studies. Importantly, we do not mean to imply that socially constructed racial identities reported by study participants are synonymous with genetic ancestry. Stratification by race may, however, capture differential social and environmental exposures within the US, which may impact the epigenome.

The meta-analysis was performed for any CHIP, *DNMT3A*, and *TET2* in White participants from FHS, CHS, and ARIC (n = 4355) and Black participants from JHS, CHS, and ARIC (n = 3841) participants, respectively, using inverse variance-weighted fixed-effects models implemented in *metagen()* function in R packages (https://rdrr.io/cran/meta/man/metagen.html). The summary statistics were used from the previous EWAS of CHIP for the ARIC and CHS cohorts.^13^ Then, cross-ancestry meta-analysis was performed for White and Black participants (n = 8,196). The meta-analysis was constrained to methylation probes passing filtering criteria in all cohorts.

Supplementary Figure 1 presents QQ plots with genomic control (GC) inflation factor (λ) to illustrate the EWAS results in each cohort and in the meta-analysis. Our observations reveal a prevalence of high inflation factors (λ >1.1) across nearly all studies. Such elevated inflation factors typically signal potential bias in the analysis process. However, it’s important to note that in cases where a significant portion of CpG sites exhibit differential methylation associated with the outcome (e.g., age and CHIP), this can contribute to the observed high λ values. Moreover, adjusting for additional PCs moderately associated with the outcome may alleviate lambda values, albeit at the expense of reduced power to detect CpGs related to the outcome. To address this, we adopted strategies consistent with those employed by the respective cohorts in previous analyses, focusing on correcting for technical variables and latent factors identified in prior studies across multiple outcomes^36–38^. Furthermore, prior to meta-analysis, we implemented additional corrections for individual study results exhibiting λ >1.5, ensuring the integrity of our findings. The statistical significance threshold was *P* <0.05/400,000 ≈ 1 × 10^−7^. A less stringent threshold, the Benjamini-corrected FDR adjusted p-value <0.05, was also used.

### Expression Quantitative Trait Methylation (eQTM) Analysis

Association tests of DNAm and gene expression were previously performed in 2115 FHS participants in the Offspring (n=686) and Third Generation (n=1429) cohorts with available whole blood DNA methylation and RNA-seq gene expression data to identify CpG sites at which differential methylation is associated with gene expression ^19^. Approximately 70,000 significant cis CpG-transcript pairs were identified at *P* <1 × 10^−7^. Cis is defined as CpGs located within 1 Mb of the transcription start site of a mRNA. When calculating the association between CpG sites and gene-level transcripts, linear regression models were used. Residualized gene expression served as the outcome and residualized DNA methylation value as the primary explanatory variable, with adjustment for age, sex, white blood cell count, blood cell fraction, platelet count, five gene expression PCs, and ten DNA methylation PCs. Through integration of CpGs and gene-level transcripts (mRNAs) from RNA-seq, mRNAs were identified that were significantly associated with each of the CpGs in cis for any CHIP and the CHIP subtypes.^19, 39^

### Pathway Enrichment Analysis

Enrichment analysis was conducted on gene sets comprising genes annotated to CpGs associated with CHIP and major CHIP subtypes, with a significance threshold of *P* <1 × 10^−7^, along with their corresponding eQTM gene sets. The DAVID Bioinformatics online tool was used for the enrichment analysis (https://david.ncifcrf.gov/home.jsp). To improve the focus of this study, only the results of Gene Ontology (GO) terms related to biological process and KEGG pathways were used. The significant threshold of FDR adjusted p-value <0.05 was used, corrected by multiple tested terms.^6^

### Cell Culture of mPB CD34+ Cells

Mobilized peripheral blood (mPB) CD34+ cells were bought from StemCell technologies or the Cooperative Center of Excellence in Hematology (CCEH) at the Fred Hutch Cancer Research Center, Seattle, USA. CD34+ cells were thawed and cultured in CD34+ expansion medium (StemSpan II (StemCell Technologies) + 10% CD34+ expansion supplement (Stemcell Technologies) + 20U/mL penicillin-streptomycin (Gibco) + 500nM UM729 (StemCell Technologies) + 750nM Stemreginin-1 (StemCell Technologies)) for 48 hours priorto editing withCRISPR-Cas9. After 48 hours, samples were electroporated with RNP complexes and seeded at 400k cells per mL. Cells were maintained between 200k - 1M cells per mL.

### CRISPR-Cas9 of mPB CD34+ Cells

Ribonucleoprotein (RNP) complexes targeting scramble, AAVS, *TET2*, ASXL-1, and DNMT3A were made by incubating Cas9 (IDT Alt-R HiFi sp Cas9 Nuclease V3) and sgRNA (IDT Alt-R Cas9 sgRNAs) at a 1:3.26 ratio. Guides for each gene are present in Supplementary Figure 7. On day 2 post thaw, mPB CD34 cells were counted and resuspended in Buffer R or GE Buffer. RNP complexes and cells were mixed and electroporated using Neon Pipette (Thermo Scientific Inc.) with the following settings: 1650V 10ms pulses 3 times. Samples were seeded in expansion media at 400k/mL.

### Assessment of Indel Formation

Genomic DNA (gDNA) was isolated and amplified with the following conditions: 95°C for 2 minutes followed by 35 cycles of 95°C for 45s, 61–62°C for 1min, 72°C for 2min with a final extension at 72°C for 5 minutes using primers towards *TET2, ASXL-1*, and *DNMT3A* (Supplementary Figure 8). PCR products were sent to GeneWiz (Azenta Life Sciences) where PCR cleanup and Sanger sequencing was performed. Indel formation was assessed using TIDE (Supplementary Figure 9).^40^

### FACS Sorting of mPB CD34+ Cells

Edited CD34+ cells were sorted at day 7 post CRISPR-Cas9 using a FACSymphony^™^ S6 Cell Sorter or a BD FACS Aria II to remove differentiated cells. Briefly, CD34+ cells were washed in cell staining buffer (Biolegend) once and stained with antibodies targeting CD34 (Biolegend), CD38 (Biolegend), and Lineage Markers (Biolegend) for 30 minutes at 4°C in the dark (Supplementary Figure 6). Antibodies from Biolegend are present in Supplementary Figure 10.

### EvoC Library Generation and Primary Methylation Analysis

DNA was extracted using Micro kits (Qiagen) from flow sorted cells from 3–5 donors. EvoC libraries were created following manufacturer instructions (Biomodal). Briefly, DNA was sheared using a Covaris LE220 and assessment of input DNA was performed using Bioanalayzer instrument (Agilent) and Qbit (ThermoFisher). Library generation was performed according to the EvoC protocol (Biomodal).

### Sequencing of EvoC Libraries

Capture of CpG sites was performed using Twist Human Methylome Panel (Twist Biosciences) and next generation sequencing was completed by using the NovaSeq 6000 (150bp paired-end reads) targeting 160M reads per sample. Biomodal pipeline version 1.1.1 was used to analyze the raw FASTQs with default settings. Briefly adaptor trimming was performed with *cutadapt*, resolution of R1 and R2 to generate single-end reads with epigenetic information, mapping onto the human genome (GRCh38), and quantification of the modification state of each CpG site.

### Comparisons between EWAS and Biomodal Data

For each sample and for each CpG, read counts from the forward and reverse strand were summed and the mC fraction calculated as the number of reads supporting mC divided by the total number of reads with modified or unmodified C (excluding reads with A, T or G). The dataset was reduced to the CpGs with significant levels of association from each EWAS analysis. For each of these CpGs, methylation difference was calculated as the difference between the average mC fraction of multiple replicates of different KO primary cells (“DNMT3A”, “TET2”, “ASXL-1”) and the average mC fraction of multiple replicates of control cells (“Scramble” or “AAVS”). Only CpGs with uncorrected p-values<0.05 (t-test) were carried forward. For each EWAS analysis (“any-CHIP”, “DNMT3A_chip”, “TET2_chip”, “ASXL1_chip” ) and for each gene-KO (“DNMT3A”, “TET2”, “ASXL-1”) the mC fraction of these CpGs was plotted against the EWAS TE, and a binomial test was used to check for enrichment in the top-right and bottom-left quadrant indicating a sign correlation between the mC fraction change induced by the KO and the EWAS TE.

### *Cis*-mQTLs

Methylation quantitative trait loci (mQTLs) – SNPs associated with DNA methylation – were identified from 4,170 FHS participants as previously reported^32^, including 4.7 million *cis*-mQTLs at *P* <2 × 10^−11^. Genotypes were imputed using the 1000 Genomes Project panel phase 3 using MACH / Minimac software. SNPs with MAF >0.01 and imputation quality ratio >0.3 were retained. Cis-mQTLs were defined as SNPs residing within 1 Mb upstream or downstream of a CpG site.

### Mendelian Randomization Analysis

In order to investigate whether differentiation methylation at CHIP-associated CpGs causally influences risk of CVD and mortality, two-sample Mendelian randomization (MR) was performed between exposures (CHIP-associated CpGs) and a list of CVD and mortality related traits as outcomes. We utilized our in-house developed analytical pipeline called MR-Seek (https://github.com/OpenOmics/mr-seek.git) to perform the analysis. The full summary statistics of different GWAS datasets were downloaded from NHGRI-EBI. The list of CVD- and mortality-related traits and list of references of those GWAS results are included in Supplementary Table 28. Previously identified *cis*-methylation quantitative trait loci (*cis*-mQTL) were utilized as instrumental variables (IVs) in the MR analysis.^21^ For each CpG site, the IVs comprised independent *cis*-mQTLs pruned for linkage disequilibrium (LD) with an r^2^ <0.01. Only one *cis*-mQTL variant with the lowest SNP-CpG p-value was retained in each LD block. For CpGs with more than one IV, Inverse-variance weighted (IVW) MR tests were conducted. Heterogeneity and MR-EGGER pleiotropy tests were employed to assess the validity of IVs. Results with a significance level of *P* <0.05 were excluded. If a CpG has only one instrumental variable (IV), the Wald MR method was applied. Significance levels of MR results were determined based on the Benjamini-Hochberg corrected FDR adjusted p-value with a threshold of <0.05.

## Data Availability

FHS datasets analyzed in the present study are available at the dbGaP repository phs000007.v32.p13 (https://www.ncbi.nlm.nih.gov/projects/gap/cgi-bin/study.cgi?study_id=phs000007.v30.p11). Genome sequencing data for JHS whole genomes are available at dbGaP phs000964 (https://www.ncbi.nlm.nih.gov/gap/). Whole genome sequencing data for the CHS cohort generated via TOPMed and CHIP somatic variant call sets are available through dbGaP phs001368. Whole exome sequencing data from the ARIC cohort are available via dbGaP accession code phs000668. DNA methylation data and phenotypic data are available via controlled access through ancillary study proposals.
